# Genome-edited *TaTFL1-5* mutation decreases tiller and spikelet numbers in common wheat

**DOI:** 10.3389/fpls.2023.1142779

**Published:** 2023-02-21

**Authors:** Jing Sun, Xiao Min Bie, Xiao Li Chu, Ning Wang, Xian Sheng Zhang, Xin-Qi Gao

**Affiliations:** National Key Laboratory of Crop Biology, College of Life Sciences, Shandong Agricultural University, Taian, China

**Keywords:** wheat, tillering, *TaTFL1*, auxin signaling, cytokinin signaling, CRISPR/Cas9

## Abstract

Tillering is a critical agronomic trait of wheat (*Triticum aestivum* L.) that determines the shoot architecture and affects grain yield. *TERMINAL FLOWER 1* (*TFL1*), encoding a phosphatidylethanolamine-binding protein, is implicated in the transition to flowering and shoot architecture in plant development. However, the roles of TFL1 homologs is little known in wheat development. CRISPR/Cas9-mediated targeted mutagenesis was used in this study to generate a set of wheat (Fielder) mutants with single, double or triple-null *tatfl1-5* alleles. The wheat *tatfl1-5* mutations decreased the tiller number per plant in the vegetative growth stage and the effective tiller number per plant and spikelet number per spike at maturity in the field. RNA-seq analysis showed that the expression of the auxin signaling–related and cytokinin signaling–related genes was significantly changed in the axillary buds of *tatfl1-5* mutant seedlings. The results suggested that wheat *TaTFL1-5s* were implicated in tiller regulation by auxin and cytokinin signaling.

## 1 Introduction

Spikes number, grains number, and grain weight are three factors for determining wheat (*Triticum aestivum* L.) grain yield (Wolde et al., 2019). Shoot architecture, defined by plant height, stem branching (tillering), phyllotaxy, and inflorescence branching, is a fundamental determinant of crop growth and yield by influencing spikes per unit of land area and grains per spike. In terms of plant development, the shoot architecture is regulated by the organization and activities of shoot apical, axillary, intercalary, and inflorescence meristems (Wang et al., 2018; Gao et al., 2019). Tillering and spikelet formation are the major determinants of spikes per unit area and grains per spike. Identifying more tillering- and spikelet formation–regulated genes is important for understanding the mechanism determining wheat yield. While several genes involved in the control of tillering have been identified, much remains to be learned about the molecular and hormonal mechanisms governing this trait. The wheat *tiller inhibition gene (tin)* encodes a cellulose synthase-like protein. The *tin* mutation inhibits wheat tillering by affecting carbon partitioning throughout the plant (Hyles et al., 2017). Additionally, wheat tae-miR156 is implicated in the regulation of tiller number by negatively regulating the expression of *SQUAMOSA-promoter binding protein-like (SPL)* genes (Liu et al., 2017; Gupta et al., 2022). Strigolactones controlling tillering in wheat have been identified. Strigolactone signaling repressor TaD53 can inhibit TaSPL activities to control tillering in wheat (Liu et al., 2017). The synthesis of strigolactones has been found to be involved in TaD27-B-regulaed tiller number in wheat (Zhao et al., 2020). PIN protein is associated with polar auxin transport (Adamowski and Friml, 2015). The wheat tiller number is significantly increased by downregulating the expression of *TaPIN1s* (Yao et al., 2021). Recently, *TIN5* encoding a homolog of a pentatricopeptide repeat protein was identified to be a new locus controlling tillering capacity in *T. urartu* (Si et al., 2022). Wheat PHYTOCHROME-INTERACTING FACTOR-LIKE (PIL) family transcription factor is involved in tillering. The overexpression of *TaPIL1* reduces the wheat tiller number (Zhang L. et al., 2022). Additionally, the overexpression of *TaCol-B5* encoding a CONSTANS-like protein in common wheat promotes tillering and increases spikelet number (Zhang X. et al., 2022).

FLOWERING LOCUS T (FT) and TERMINAL FLOWER 1 (TFL1), two homologs of the phosphatidylethanolamine-binding protein family, are implicated in flowering transition and inflorescence architecture in plant development. *TFL1* controls inflorescence meristem identity and retards shoot apical meristem transition to the reproductive phase (Kaneko-Suzuki et al., 2018; Zhu et al., 2020). Besides flowering induction, *TFL1* is also implicated in determining inflorescence architecture by promoting the indeterminacy of the inflorescence meristem (Bradley et al., 1997). In rice, the knock-down expression of *RICE CENTRORADIALISs (RCNs)*, rice homologs of *TFL1*, reduces panicle size and branches (Liu et al., 2013). A barley mutant in the *TFL1* homolog *HvCEN* decreases the number of spikelet and tiller (Bi et al., 2019). In contrast, the ectopic expression of *TFL1* homolog genes in rice and maize increases inflorescence branching and complexity (Nakagawa et al., 2002; Danilevskaya et al., 2010). Tomatoes with the dominant allele of SELF-PRUNING (SP, a TFL1 homolog) exhibit over growth with more inflorescences and fruits (Jiang et al., 2013). Thus, TFL1 homologs in crops are involved in shoot architecture. The common wheat genome encodes nine putative TFL1-like proteins (Dong et al., 2020). The overexpression of *TaTFL1-2D* (*TaPEBP20*) in wheat enhances the production of spikelet, indicating that *TaTFL1-2D* modifies inflorescence architecture (Wang et al., 2017). In this study, the expression patterns, localizations, and functions of *TaTFL1-5s* (*TaTFL1-5A*, TraesCS5A02G128600; *TaTFL1-5B*, TraesCS5B02G127600; and *TaTFL1-5D*, TraesCS5D02G136300) were analyzed for their characterization in common wheat development.

## 2 Materials and methods

### 2.1 Plant materials and growth conditions

The experimental materials is wheat cultivar Fielder (Liang et al., 2022) in this study, which is provided by Dr. Xingguo Ye (Institute of Crop Sciences, Chinese Academy of Agricultural Sciences, Beijing, China). The wild-type and mutant seeds were sowed in the Experimental Station of Shandong Agricultural University (China). Each experimental line was planted with 25-cm in-row spacing and 12.5-cm plant-to-plant spacing.

### 2.2 Quantitative real-time polymerase chain reaction analysis

Total RNAs were extracted from different wheat (Fielder) tissues using an Ultrapure RNA Kit (CoWin Biosciences, Beijing, China), which was used for the reverse transcription with a FastQuant RT kit (Tiangen, Beijing, China). qRT-PCR was performed using LightCycler (Roche Diagnostics, USA). 2×SuperReal PreMix Plus (with SYBR Green) (Tiangen, Beijing, China) was used as the premix and the diluted cDNA was used as the template. PCR was carried out as follows: pre-denaturation at 95°C for 900 s, followed by 45 cycles of 95°C for 10 s (denaturation), 58°C for 20 s (anneal), 72°C for 20 s (extension), and the final extension at 95°C for 10 s, 65°C for 60 s, and 97°C for 1 s. delta-Ct and melting curve were analyzed by using software LightCycler 96. The expression of *TaActin* or *TaTubulin* was used for RNA calibration. All the qRT-PCR experiments were repeated more than three times. The primers used for qRT-PCR are listed in **Supplementary Table S1**.

### 2.3 *In situ* hybridization

Fresh wheat materials in different stages were harvested and immediately fixed in FAA solution (10% formalin, 5% acetic acid, and 50% ethanol), embedded in paraffin, and finally sectioned at 8 μm. The full-length *TaTFL1-5s* were used as the template to synthesize the probes that were labeled with digoxigenin using a DIG RNA Labeling Kit (SP6/T7, Roche). Then, the probes were hydrolyzated for the next use. The reaction and the analysis of hybridized signals were performed as Zhao et al. (2020). After gradient dehydration and rehydration using xylene and ethanol, the samples were incubated with hybrid solution containing the probes at 42°C overnight. Anti-digoxigenin antibodies were used to detect the hybridization signals. The primers used are listed in **Supplementary Table S1**.

### 2.4 Subcellular localization analysis

To construct 35S::*TaTFL1-5*s-*GFP* for the subcellular localization analysis of TaTFL1-5s, the full-length CDS sequences of *TaTFL1-5*s were cloned into pMDC43 vector, which was placed in the frame upstream of *GFP* sequence. The construction was sequenced to make sure it was correct. The localization of TaTFL1-5s-GFP was analyzed in *Nicotiana benthamiana* (tobacco) leaves by transient expression, as described by Xiong et al. (2019). The epidermis of infected leaves was observed under a confocal laser scanning microscope (LSM880, Zeiss, Germany). GFP signal was visualized using an excitation wavelength of 488nm and emission wavelength of 505-520nm. The primers used are listed in **Supplementary Table S1**.

### 2.5 Vectors construction for CRISPR/Cas9 mediated editing and wheat transformation

The mutants of *TaTFL1-5s* were created using clustered regularly interspaced short palindromic repeats/CRISPR-associated protein 9 (CRISPR/Cas9) genome editing technology (Wang et al., 2015). E-CRISP (http://www.e-crisp.org/E-CRISP/) was used to design the two target sites. The primers *TaTFL1*-5s-F, *TaTFL1*-5s-F0, *TaTFL1*-5s-R, and *TaTFL1*-5s-R0 (**Table S1**) was used for PCR amplification and pCBC-MT1T2 was used as the template. The PCR fragment was purified and inserted into expression vector pBUE411-Cas9, as described by Li et al. (2022). The callus cultured from immature embryo of Fielder was used for transformation by *Agrobacterium tumefaciens*–mediated method (PureWheat), as described by Medvecká and Harwood (2015). The regenerated plants were used for further analysis. The primers used are listed in **Supplementary Table S1**.

### 2.6 Identification of the edited mutant lines

The genome of wheat leaves was extracted using CTAB buffer (1%(w/v)CTAB, 50mM Tris (pH 8.0), 10mM EDTA, 0.7M NaCl) (Saghai-Maroof et al., 1984). The genome-specific primer set *TaTFL1*-JD-5AF/*TaTFL1*-JD-5AR was used for the amplification of gRNA1 and gRNA2 of A subgenome; *TaTFL1*-JD-5BF/*TaTFL1*-JD-5BR was designed to amplify gRNA1 and gRNA2 of B subgenome; and *TaTFL1*-JD-5DF/*TaTFL1*-JD-5DR was designed to amplify gRNA1 and gRNA2 of D subgenome (**Table S1**). The PCR products were sequenced. The sequencing results were aligned with the reference sequences to screen *tatfl1-5* mutant plants.

### 2.7 Off-target analysis in the edited mutant lines

The potential off-target sites were selected to investigate the off-target effect predicted using WheatOmics (http://202.194.139.32/blast/blast.html) based on sequence similarity. Specific primes of the potential off-targets were used for PCR, and the products were sequenced to test the off-target. The primer sets are listed in **Supplementary Table S1**.

### 2.8 RNA sequencing and data analysis

The axillary buds of *tatfl1-5* mutant 3-week seedlings was used for the extraction of the total RNA using an OminiPlant RNA Kit (DNase I) (CoWin Biosciences, MA, USA) from. NEBNext Ultra RNA Library Prep Kit for Illumina (E7530) (New England Biolabs, MA, USA) was used to generate the sequencing libraries, and the Illumina sequencing platform was used for RNA libraries sequence by Qingdao Ouyi Biotechnology Co. Ltd (Qingdao, China). The RNA-seq were performed three biological replicates and the data was deposited in Gene Expression Omnibus datasets (accession number: GSE218387) at National Center for Biotechnology Information. The wheat reference genome and genes (http://plants.ensembl.org/Triticum_aestivum/Info/Index) was used for the mapping of the filtered clean reads. The gene expression level was normalized using the software DESeq2. The KEGG pathway (http://www.genome.jp/kegg/) and GO (http://geneontology.org/) of differentially expressed genes (DEGs) were analyzed.

### 2.9 Construction of phylogenetic tree

The TFL1 protein family identification and phylogenetic tree construction were performed according to the description by Sun et al. (2020). The *Arabidopsis* TFL1 was used for BLAST homology search. The reference genomic sequence were retrieved from the Ensemble Plants (http://plants.ensembl.org/). The phylogenetic tree was constructed using MEGA6.0 (Maximum likelihood) (https://www.megasoftware.net/).

## 3 Results

### 3.1 Expression patterns of *TaTFL1-5* genes and subcellular localization of TaTFL1-5s

**Figure 1:**
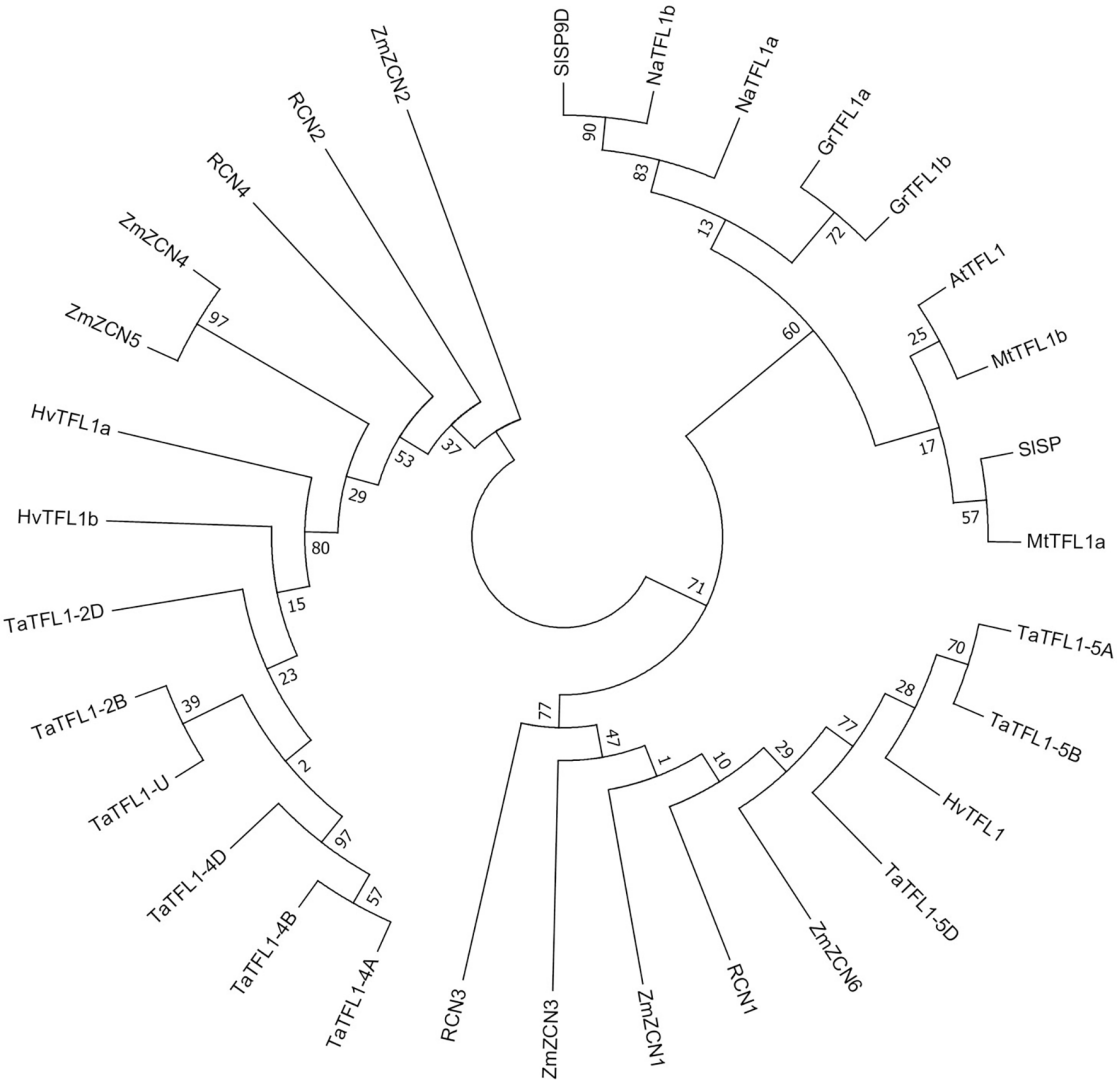
Phylogenetic analysis of TFL1 and its homologs in plant species. The rootless phylogenetic tree of TFL1 and its homologs from *Hordeum vulgare*, *Triticum aestivum*, *Oryza sativa*, *Zea mays*, *Solanum lycopersicum*, *Nicotiana attenuate*, *Gossypium raimondii* and *Medicago truncatula* was constructed by using MEGA 6.0 (**Table S2**).

**Figure 2:**
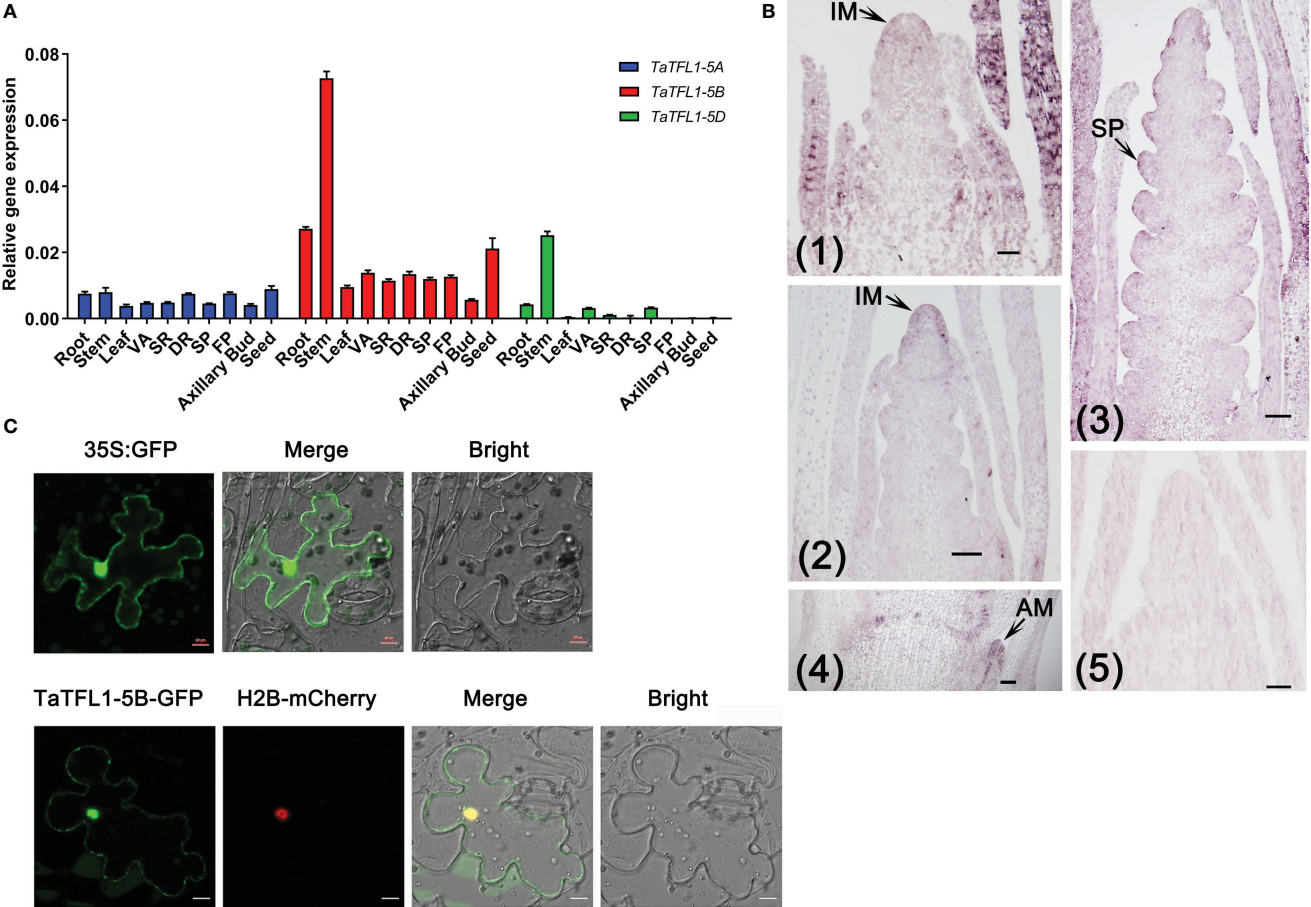
Expression patterns of *TaTFL1*-5s and the localization of TaTFL1-5B. **(A)** qRT-PCR analysis of the relative expression levels of *TaTFL1*-5s in different tissues of wheat development. DR, Shoot apex at the double ridge stage; FP, Shoot apex at the floret primordium stage; SP, Shoot apex at the spikelet primordium stage; SR, Shoot apex at the single ridge stage; VA, Vegetative apex; **(B)** RNA *in situ* hybridization analysis of the expression patterns of *TaTFL1*-5s at the early stage of spike development. (1) Longitudinal section of the shoot apex at the single ridge stage; (2) Longitudinal section of the shoot apex at the double ridge stage; (3) Longitudinal section of the young spike; (4) Longitudinal section of the young stem showing the axillary meristem; (5) Sense probe control. AM, axillary meristem; IM, Inflorescence meristem; SP: spike primordium. Bars, 100 µm. **(C)** Co-localization of TaTFL1-5B with H2B visualized using transient expression of 35S::*TaTFL1-5B-GFP* and 35S::*H2B-mCherry* in tobacco leaves. The co-localization signal was tested at 50 h after infection. The expression of 35S:*GFP* was used as the control. Bars, 10 µm.

A total of 3, 1, 9, 4, 6, 2, 2, 2 and 2 *TFL1-like* genes were identified in *Hordeum vulgare*, *Arabidopsis thaliana*, *Triticum aestivum*, *Oryza sativa*, *Zea mays*, *Solanum lycopersicum*, *Nicotiana attenuate*, *Gossypium raimondii* and *Medicago truncatula*, respectively. A rootless phylogenetic tree of TFL1 and its homologs was constructed (**Figure 1** and **Table S2**). The tree showed *TaTFL1-5*s have the closest phylogenetic relationship with barley *HvTFL1* (**Figure 1**). Then, we characterized the expression patterns of *TaTFL1-5s* in various tissues and organs (roots, stems, leaves, axillary buds, seeds, and shoot apex in different stages) of wheat by qRT-PCR analysis using *TaActin* expression as an internal control. The results revealed that *TaTFL1-5s* showed ubiquitous expression in wheat (**Figure 2A**). Additionally, *TaTFL1-5B* showed a higher expression level compared with *TaTFL1-5A* and *TaTFL1-5D* (**Figure 2A**); the highest expression level of *TaTFL1-5B* was found in the stem (**Figure 2A**). In contrast, *TaTFL1-5D* expression was hardly detectable in leaves and shoot apex in the double ridge and floret primordium stages, axillary buds, and seeds (**Figure 2A**). RNA *in situ* hybridization analysis revealed that *TaTFL1-5s* expressed in the inflorescence meristems in the single-ridge and double-ridge stages and spikelet primordium in young spikes. In addition, their expression was detected in axillary meristem (**Figure 2B**). To find the reason for the highest expression of *TaTFL1-5B* in wheat, we analyzed the *cis*-elements in the promoter sequences (2000 bp upstream of the start codon) of *TaTFL1-5s* genes. There are two cis-elements in the promoter region of *TaTFL1-5B*, Gap-box (CAAATGAA[A/G]A) and Box III (atCATTTTCACt), which do not present in those of *TaTFL1-5A* and *D.* Gap-box and Box III confer light responsiveness to gene (Weisshaar et al., 1991; Conley et al., 1994; Park et al., 1996), which might be important for the high expression of *TaTFL1-5B*.

The subcellular localization was determined using the transient expression of 35S::*TaTFL1-5A/B/D-GFP* vector in epidermal cells of tobacco and H2B-mCherry as a marker of nucleus. The expression of 35S:*GFP* was used as the control. The fluorescence signal of the TaTFL1-5A/B/C-GFP fusion protein was observed in both the nucleus and the cytoplasm, as shown in **Figure 2C** and **Figure S1**.

### 3.2 CRISPR/Cas9-mediated *tatfl1-5s* mutation in wheat

**Figure 3:**
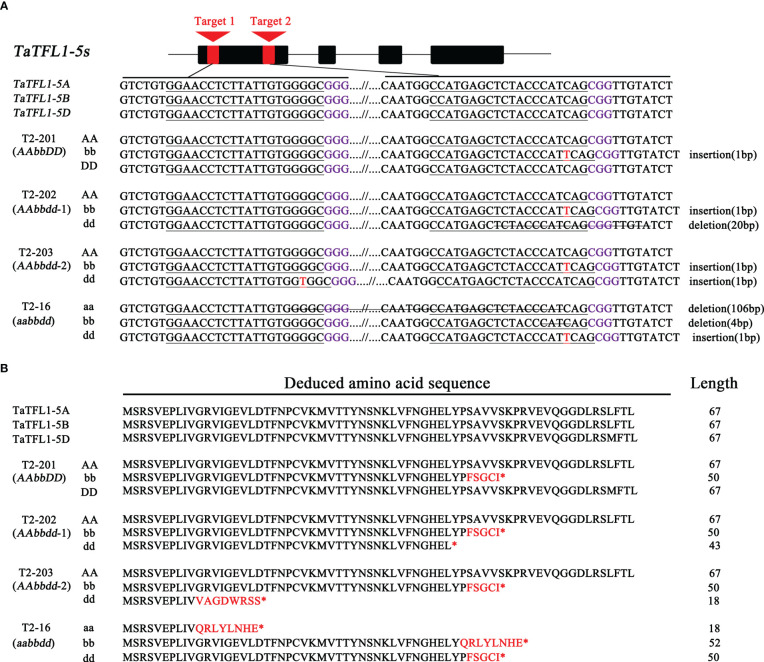
CRISPR/Cas9-mediated targeted mutagenesis of *TaTFL1-5s* gene in wheat. **(A)** Gene structure of *TaTFL1-5s* and genotypes of different mutant lines obtained by CRISPR/Cas9 strategy. The PAM motifs are highlighted in purple, and the target sequences are underlined. Insertions are highlighted in red, and the deletions are indicated with strikethrough. Line T2-201 contained a homozygous mutation around target 2 in B genome (an insertion of 1 bp). Line T2-202 had homozygous mutations around target 2 in B genome (an insertion of 1 bp) and in D genome (a deletion of 20 bp). Line T2-203 had homozygous mutations around target 2 in B genome (an insertion of 1 bp) and around target 1 in D genome (an insertion of 1 bp). In Line T2-16, the homozygous mutations are around target 1 and 2 in A genome (a deletion of 106 bp), around target 2 in B genome (a deletion of 4 bp) and around target 2 in D genome (an insertion of 1 bp). **(B)** The mutant amino acid sequence in different homozygous mutant lines compared to wild type (WT) Fielder. Substitutions of amino acid residues are shown by red-colored text; “*” indicates that translation is terminated.

Two guide RNAs targeted to two shared sequences (Target 1 and Target 2 in **Figure 3A**) of *TaTFL1-5s* were designed for generating knockout lines using CRISPR/Cas9 genome editing technology (Wang et al., 2015) to dissect the roles of *TaTFL1-5s* in wheat development. The sgRNA was synthesized and cloned into an expression vector pBUE411-Cas9. Then, the *Agrobacterium tumefaciens*–mediated infection was used for the vector transformation in spring wheat variety Fielder (Medvecká and Harwood, 2015). The identified T1 mutant plants were selfed, and the resulting plants (T2) were used to identify the genotypes. Primers specifically amplifying *Cas9* were used to identify the transgene-free lines. Four transgene-free mutant lines of T2, including one *AAbbDD* line (T2-201), two *AAbbdd* lines (T2-202 and T2-203), and one *aabbdd* line (T2-16), were obtained by PCR analysis with primers specific to targeting site and sequencing (**Figure 3A**). In these mutants, the *a*, *b*, and *d* indicated the null mutations of *TaTFL1-5* in the three subgenomes, respectively. In the *AAbbDD* line (T2-201), 1-bp insertion at the targeting site 2 in the B subgenome was identified (**Figure 3A**). The *AAbbdd-1* line (T2-202) had 1-bp insertion and 20-bp deletion at the targeting site 2 in B and D subgenomes, respectively (**Figure 3A**). The *AAbbdd-2* line (T2-203) had 1-bp insertions at the targeting site 2 and site 1 in B and D subgenomes, respectively (**Figure 3A**). The *aabbdd* line (T2-16) had 106-bp deletion, 4-bp deletion, and a 1-bp insertion at the targeting site 2 in A, B, and D subgenomes, respectively (**Figure 3A**). The mutations in these lines led to pseudogenes with premature stop codons (**Figure 3B**). In the potential off-target regions, no mutations were found in these mutants (**Figure S2**). These mutant lines were selfed, and the resulting plants (T3) were identified using specific primers for amplifying and sequencing to confirm the genotype. The mutations observed in T2 could be stably transmitted to the subsequent generation (T3). Then, the T3 generation was used for subsequent examination.

### 3.3 *tatfl1-5* mutants showed decreased tillers and spikelets

**Figure 4:**
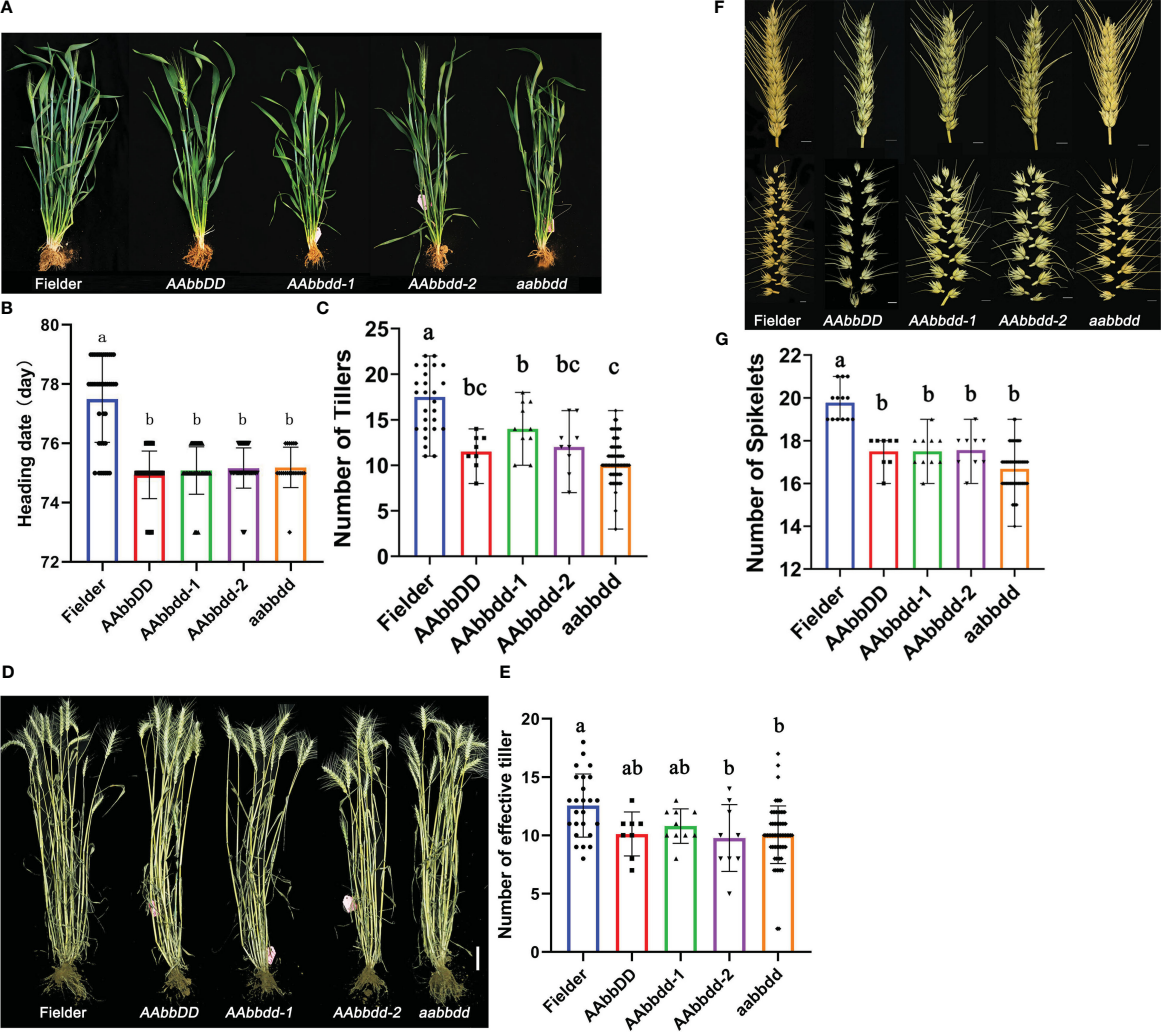
Phenotypes of wheat plants with *TaTFL1-5s* edition. **(A)** Appearance of plants of Fielder and the mutant lines at heading stage. Bar, 100 mm. **(B)** Statistics analysis of the heading data of Fielder and the mutant lines. Significant differences are denoted with distinct letters (Tukey’s *post hoc* test, *p* < 0.05). The error bars indicate the standard deviation of triplicate values. **(C)** Statistics analysis of tiller number of Fielder and the mutant lines at heading stage. Significant differences are denoted with distinct letters (Tukey’s *post hoc* test, *p* < 0.05). The error bars indicate the standard deviation of triplicate values. **(D)** Appearance of plants of Fielder and the mutant lines at maturity. Bar, 100 mm. **(E)** Statistics analysis of effective tiller number of Fielder and the mutant lines at maturity. Significant differences are denoted with distinct letters (Tukey’s *post hoc* test, *p* < 0.05). The error bars indicate the standard deviation of triplicate values. **(F)** Appearance of spike (up panel) and spikelet (down panel) of Fielder and the mutant lines at mature stage. Bars, 10 mm. **(G)** Statistics analysis of the spikelet number per spike of Fielder and the mutant lines. Significant differences are denoted with distinct letters (Tukey’s *post hoc* test, *p* < 0.05). The error bars indicate the standard deviation of triplicate values.

The *tatfl1-5* wheat lines (*AAbbDD*, *AAbbdd-1*, *AAbbdd-2*, and *aabbdd*) were sowed in the field to study the vegetative and reproductive development phenotypes. Then, the plant architecture of wheat mutants was investigated in detail. In the vegetative growth stage, *tatfl1-5* mutant plants did not show any visible difference from the wild-type Fielder plants under field conditions. We found that the heading time of the *tatfl1-5* mutant was significantly earlier compared with the Fielder (**Figures 4A, B**). In the heading stage (75 days after sowing), all *tatfl1-5* mutant plants (10–14 tillers per plant) had decreased number of tillers compared with the Fielder (16.9 tillers per plant) (**Figures 4A, C**). The statistical analysis revealed that the tiller number per plant significantly decreased in the mutant lines compared with the Fielder (**Figure 4C**). At maturity, wild-type Fielder plants could create an average of 12.5 effective tillers per plant (**Figures 4D, E**). Compared with the wild type, the plants of *AAbbdd-2* and *aabbdd* mutant lines had fewer effective tillers, 9.8 and 10.1 tillers per plant, respectively (**Figures 4D, E**). Additionally, the average spikelet number per spike also significantly decreased in these mutant lines (17–17.5 spikelets per spike) compared with the Fielder (19.8 spikelets per spike) (**Figures 4F, G**).

### 3.4 Transcripts regulated in the axillary bud initiation stage in *tatfl1-5* mutant

**Figure 5:**
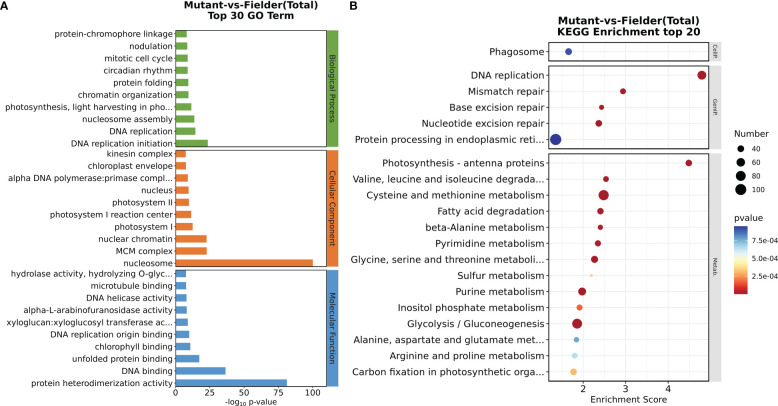
GO and KEGG analysis of the DEGs. **(A)** Classification of the DEGs by GO analysis performed on the website GENE ONTOLOGY (). The top 30 enriched terms are shown. **(B)** Scatter plots of KEGG pathway-enrichment statistics performed on the website Kyoto Encyclopedia of Genes and Genomes (). The top 20 enriched pathways are shown. The vertical axis represents the pathway categories. The size of the “number” represents the number of genes clustered in a singling pathway. The bigger the point size, the more genes in the pathway.

A comparative transcriptome analysis was performed through RNA sequencing (RNA-seq) to illuminate further the molecular basis for the tiller number difference between the wild-type Fielder and *tatfl1-5* mutant (*aabbdd*). The axillary buds of *tatfl1-5* mutant 10-day seedlings were collected for analysis and those of Fielder as the control. Six RNA libraries were sequenced with three biological replicates each for Fielder and *tatfl1-5* mutant. The clustering analysis and the principal component analysis revealed that the gene expression was reproducible among the biological replicates (**Figure S3**). per library produced about 40.57–48.77 million raw reads. After filtration, about 38.9–46.8 million clean data per library were obtained (**Table S3**). Then, DEGs were screened out using twofold change as a basis of *q*-value <0.05. Compared with the wild-type Fielder, 4,394 were upregulated and 3,160 were downregulated in the identified 7,554 DEGs in the *tatfl1-5* mutant (**Figure S4** and **Table S4**). To test the validity of sequencing data, the expression of eight selected-DEGs were analyzed by qRT-PCR. The results were consistent with those of RNA sequencing, which verified that the sequencing data is credible (**Figure S5**). GO enrichment analysis was also performed to gain more insights about DEGs. The genes associated with DNA replication, nucleosome assembly, protein folding, and chromatin organization were significantly enriched (**Figure 5A**). KEGG analysis is helpful for the understanding of the molecular interactions among the DEGs. **Figure 5B** presents the top 20 overrepresented pathways, in which the genes involved in DNA replication and photosynthesis-antenna proteins were enriched.

**Figure 6:**
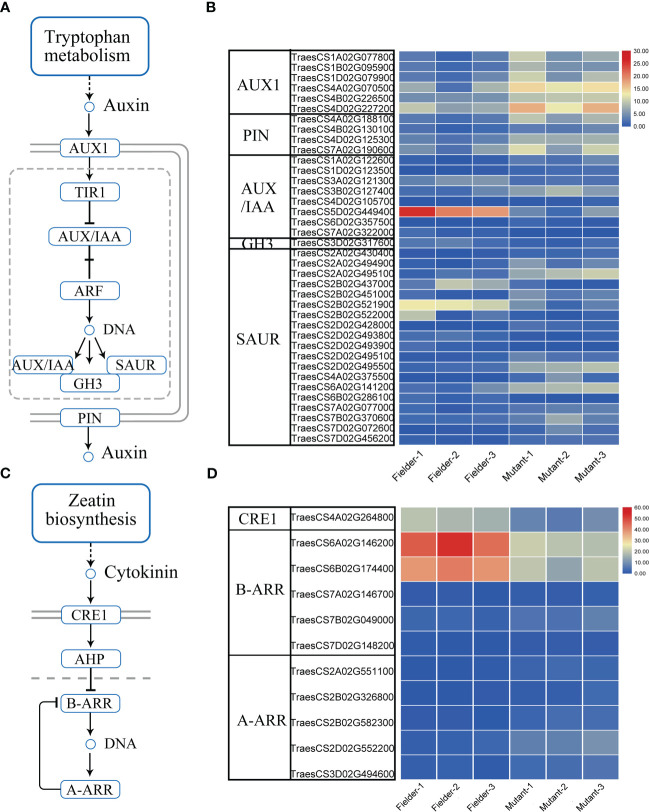
Auxin and cytokinin signaling pathways and the DEGs in these signaling pathways. **(A)** Identified auxin signaling pathway in plant. **(B)** Heat map displaying the relative expression levels of the key auxin signaling-related genes in the DEGs. **(C)** Identified cytokinin signaling pathway in plant. **(D)** Heat map displaying the relative expression levels of the key cytokinin signaling-related genes in the DEGs.

Additionally, the hormone signal processes were enriched in the DEGs. Auxin is implicated in inhibiting the outgrowth of axillary buds. The reduction of auxin at leaf axils is required for axillary meristem (AM) formation during vegetative development (Wang Q. et al., 2014). Auxin efflux is a prerequisite for the auxin depletion of leaf axil and AM initiation (Wang Q. et al., 2014). The polar localization of auxin transporters is required for auxin transport (Adamowski and Friml, 2015). The AUXIN1/LIKE-AUX1 (AUX1/LAX) family is the major auxin influx carrier, whereas the PIN-FORMED (PIN) family is used for auxin efflux. The downregulation of *TaPIN1s* increases the tiller number (Yao et al., 2021). This study showed that the expression of wheat *AUX1* and *PIN* was upregulated remarkably in the *tatfl1-5* (**Figures 6A, B**). Our results revealed that many early auxin-responsive genes, such as *AUXIN/INDOLE ACETIC ACID* (*AUX/IAA*), *Gretchen Hagen 3* (*GH3*), and *SMALL AUXIN UP RNA* (*SAUR*) family genes (Hagen and Guilfoyle, 2002), showed significant changes in expression in the *tatfl1-5* mutant compared with those in the Fielder (**Figures 6A, B**). Auxin and cytokinin play antagonistic functions in regulating the outgrowth of AM. Cytokinin perception and signaling promote AM initiation (Wang Y. et al., 2014). *Arabidopsis* Cytokinin Response 1 (CRE1) is a cytokinin receptor, which perceives cytokinin and transmits the signal *via* a multistep phosphorelay (Inoue et al., 2001). *Arabidopsis* response regulator (ARR) proteins are the phosphorelay targets. Type-A ARRs are the negative regulators of cytokinin signaling, whereas Type-B ARRs play positive roles in cytokinin signaling that regulate the transcription of lots of genes, such as the type-A *ARR*s (To et al., 2007). The present study showed that a wheat *CRE1* homolog gene was downregulated in the *tatfl1-5* mutant compared with that in the Fielder (**Figures 6C, D**). Additionally, the expression of lots of wheat ARR genes were significantly changed in the *tatfl1-5* mutant (**Figures 6C, D**).

*SPL* gene family is plant-specific and encodes transcription factor. Several *SPL* genes have been identified from rice, wheat, and *Arabidopsis*, which control tillering and AMs (Jiao et al., 2010; Miura et al., 2010; Tian et al., 2014; Liu et al., 2017; Gupta et al., 2022). Eight genes (TraesCS2B02G250900, TraesCS2B02G432700, TraesCS2D02G410700, TraesCS2D02G502900, TraesCS5A02G265900, TraesCS7A02G246500, TraesCS7B02G144900, and TraesCS7D02G245200) encoding wheat SPL homologs showed significantly increased expression in *tatfl1-5* mutant (**Table S4**). PHYTOCHROME-INTERACTING FACTOR-LIKE (PIL) family transcription factors are newly identified repressors of tillering in cereal crops (Zhang L. et al., 2022). Wheat TaPIL1 directly physically interacts with wheat TaSPL3/17, and the overexpression of *TaPIL1* reduces the wheat tiller number. Our study found that wheat *TaPIL1* gene (TraesCS5A02G376500 and TraesCS5D02G386500) expression was upregulated in the *tatfl1-5* mutant (**Table S4**). These results indicated that *TaTFL1*-5s were involved in tillering by regulating the expression of *SPL* and *PIL1* in wheat.

## 4 Discussion

The shoot architecture of wheat is a fundamental determinant of growth and yield by influencing spikes per unit of land area and grains per spike. The tiller number per plant contributes to the formation of yield. Understanding the molecular mechanisms determining tillering is usful for crop genetic improvement. To date, only a few tillering-regulated genes have been identified in wheat. This study found that *TaTFL1-5s* participated in wheat tillering and spikelet formation. Tillers are derived from axillary bud primordium, which formed at the axil of the leaf primordium (Shang et al., 2021). Tiller development is related with two successive stages: formation of the axillary meristem and growth of the axillary buds. The spikelet is derived from the lateral spikelet meristem. Additionally, the overexpression of *TaTFL1-2D* in wheat up-regulates the spikelet number (Wang et al., 2017). Thus, *TaTFL1* were implicated in the activity regulation of both shoot axillary meristems and lateral spikelet meristems. The overexpression of *TaCol-B5* in common wheat leads to increase tillering and spikelet number (Zhang X. et al., 2022). Thus, the regulation of tillering and spikelet formation shares some similar molecular mechanism.

FT and TFL1 physically interact with FD *via* 14-3-3 proteins, and hence are implicated in transcriptional regulation (Zhu et al., 2020). It has been found that FT is a transcriptional co-activator while TFL1 act as a co-repressor. FT and TFL1 play opposite roles depending on their competition bounding of FD. This study identified 4,394 upregulated genes in the axillary buds of the *tatfl1-5* mutant, which was much more than the downregulated ones, indicating that TFL1 served as a repressor in gene expression regulation. Our analysis revealed that the genes associated with DNA replication, nucleosome assembly, protein folding, chromatin organization, and hormone signaling were significantly enriched in the DEGs of the axillary buds during wheat tillering. Barley *HvCEN* is the homolog of *TFL1*. The *hvcen* mutant of barley showed a reduction in spikelet number per spike (Bi et al., 2019). The main shoot apex transcriptome analysis revealed that the genes with functions in chromatin remodeling activities and cytokinin signaling were enriched (Bi et al., 2019). These results indicated that a similar molecular mechanism was adopted by TFL1 to regulate tillering and spikelet formation. In the DEGs of the *tatfl1-5* mutant, the genes related to auxin signaling and cytokinin signaling were enriched. For example, the polar auxin transport-related genes and auxin-regulated genes all showed changes in expression. Additionally, the expression of the cytokinin receptor gene *CRE1* homolog and a large number of *ARR* genes was changed in the *tatfl1-5* mutant. During AM outgrowth, auxin and cytokinin play opposite functions. That is to say, auxin has inhibition roles, whereas cytokinins enhancing bud formation (Wang Y. et al., 2014). Thus, TFL1 may be involved in both auxin signaling and cytokinin signaling in regulating wheat tillering.

*TFL1* plays negative role in controlling *Arabidopsis* flowering time and regulates inflorescence architecture (Simon et al., 1996). Additionally, *LpTFL1* in ryegrass, *RCN*s in rice, *ZEA CENTRORADIALISs* (*TFL1* homologs in maize) and *HvCEN* in barley also play similar roles (Jensen et al., 2001; Nakagawa et al., 2002; Danilevskaya et al., 2010; Bi et al., 2019). In this study, we found that the heading time of the *tatfl1-5* mutant was earlier than that of the Fielder and the number of the tiller and spikelet decreased compared with the control, indicating the roles of *TFL1* are conserved in controlling flowering time and shoot architecture. In wheat, lots of genes identified in controlling flowering time are implicated in the regulation of the number of the tiller and spikelet, such as *TaCol-B5*, *FT-B2*, *AGAMOUS-LIKE6*, *VERNALIZATION 1*, *SHORT VEGETATIVE PHASEs*, and *WHEAT ORTHOLOG OF APO1* (Li et al., 2019; Gauley and Boden, 2021; Li et al., 2021; Kong et al., 2022; Kuzay et al., 2022; Zhang X. et al., 2022). The changes of flowering time might result from the alteration of the transition time from the vegetative apical meristem to the reproductive apical meristem in wheat development, which leads to the changes of the differentiation duration of tiller and spikelet, as suggested in *Arabidopsis* (Hanano and Goto, 2011).

## Data availability statement

The data presented in the study are deposited in the in Gene Expression Omnibus datasets at National Center for Biotechnology Information, accession number GSE218387.

## Author contributions

XZ and X-QG designed the project. JS, XB, XC, and NW conducted the experiments. X-QG wrote the manuscript. All authors contributed to the article and approved the submitted version.
