# Supplementary material for: Genome-edited TaTFL1-5 mutation decreases tiller and spikelet numbers in common wheat
Source: Front Plant Sci. 2023 Feb 21;14:1142779. doi: 10.3389/fpls.2023.1142779 (PMC9989183; doi:10.3389/fpls.2023.1142779)
Supplement: Supplementary file 1 [file DataSheet_1.docx]

**Supplemental Figures**


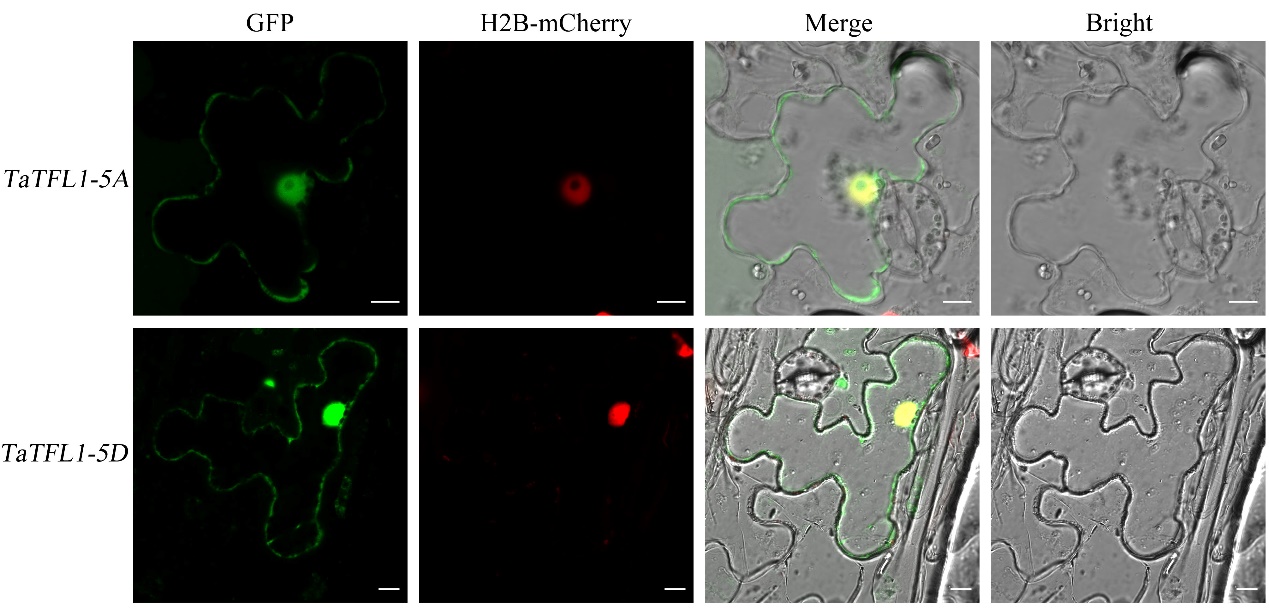


**Supplementary Figure S1 Co-localization of TaTFL1-5s with H2B-mCherry visualized using transient expression of 35S::*TaTFL1-5s-GFP* and 35S::*H2B-mCherry* in tobacco leaves.**

The co-localization signal was tested at 30 h after infection. Bars, 10 µm.


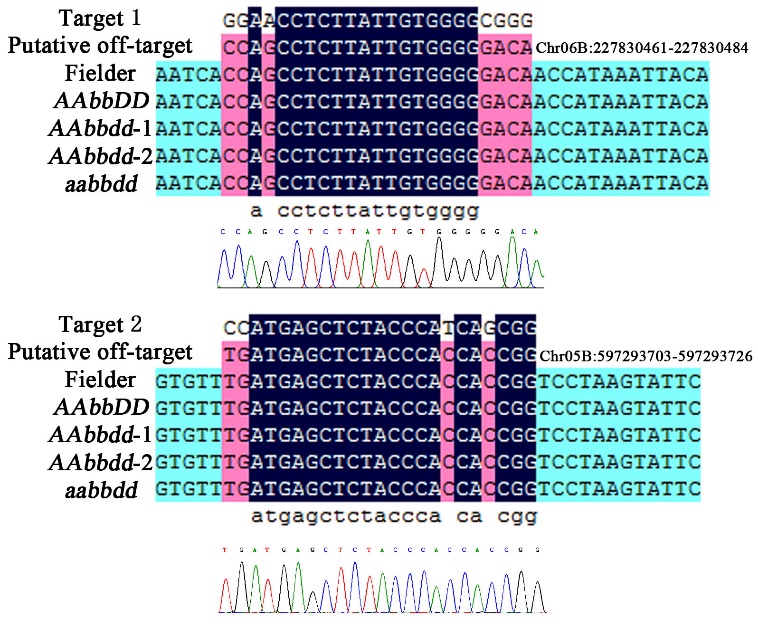


**Supplementary Figure S2 Analysis of the potential off-target sites for *TaTFL1-5* editing.**

The target sequences, the putative off-target sequences and the sequenced genomic sequences in the Fielder and the mutant lines were listed. The sequence alignment was performed using software DNAMAN 8. The lower panel of each image is the sequencing result of genome showed by software Chromas 2.5.


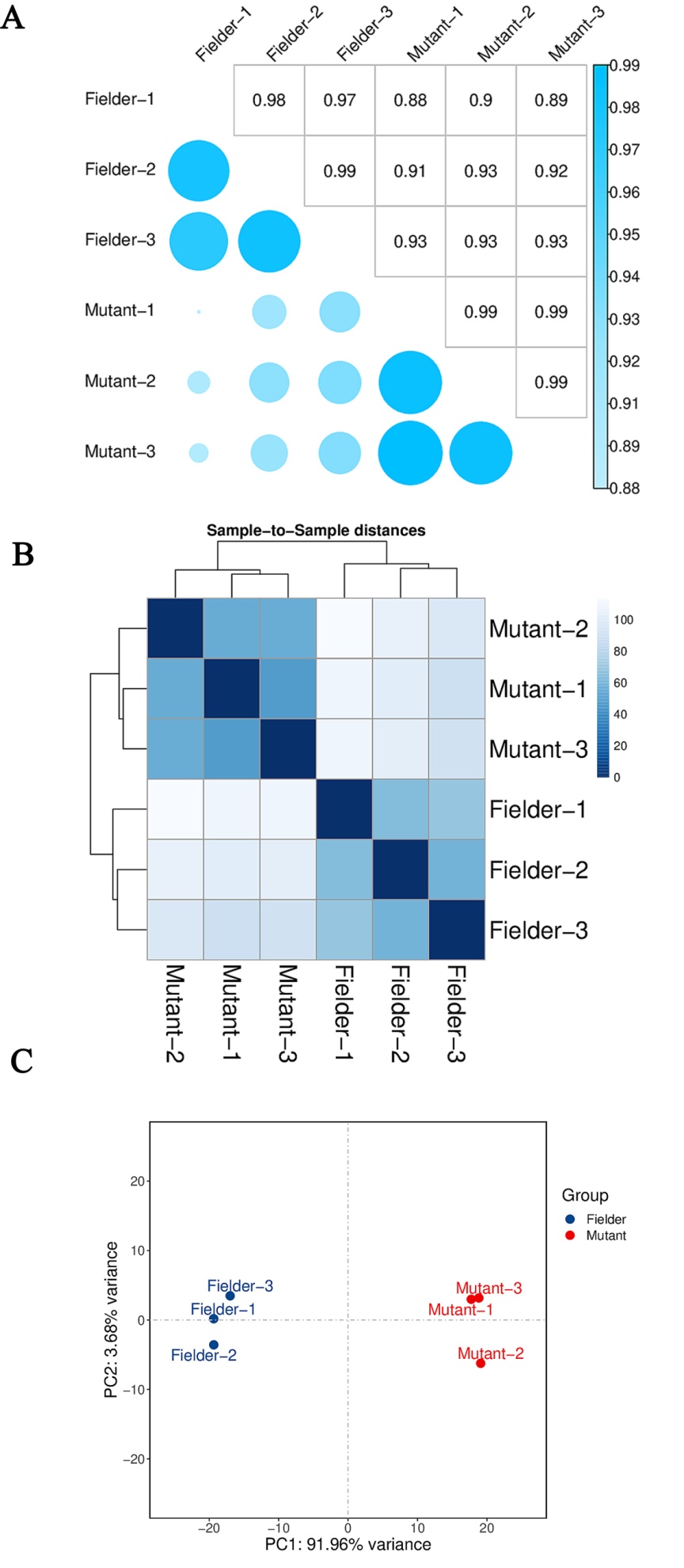


**Supplementary Figure S3 Correlation coefficients, clustering analysis and principle component analysis of the RNA-seq samples.**

A Heat map of the correlation coefficients between the samples.

B Heat map of the sample clustering analysis for checking batch effects and their similarity.

C Principle component analysis for gene expression pattern.


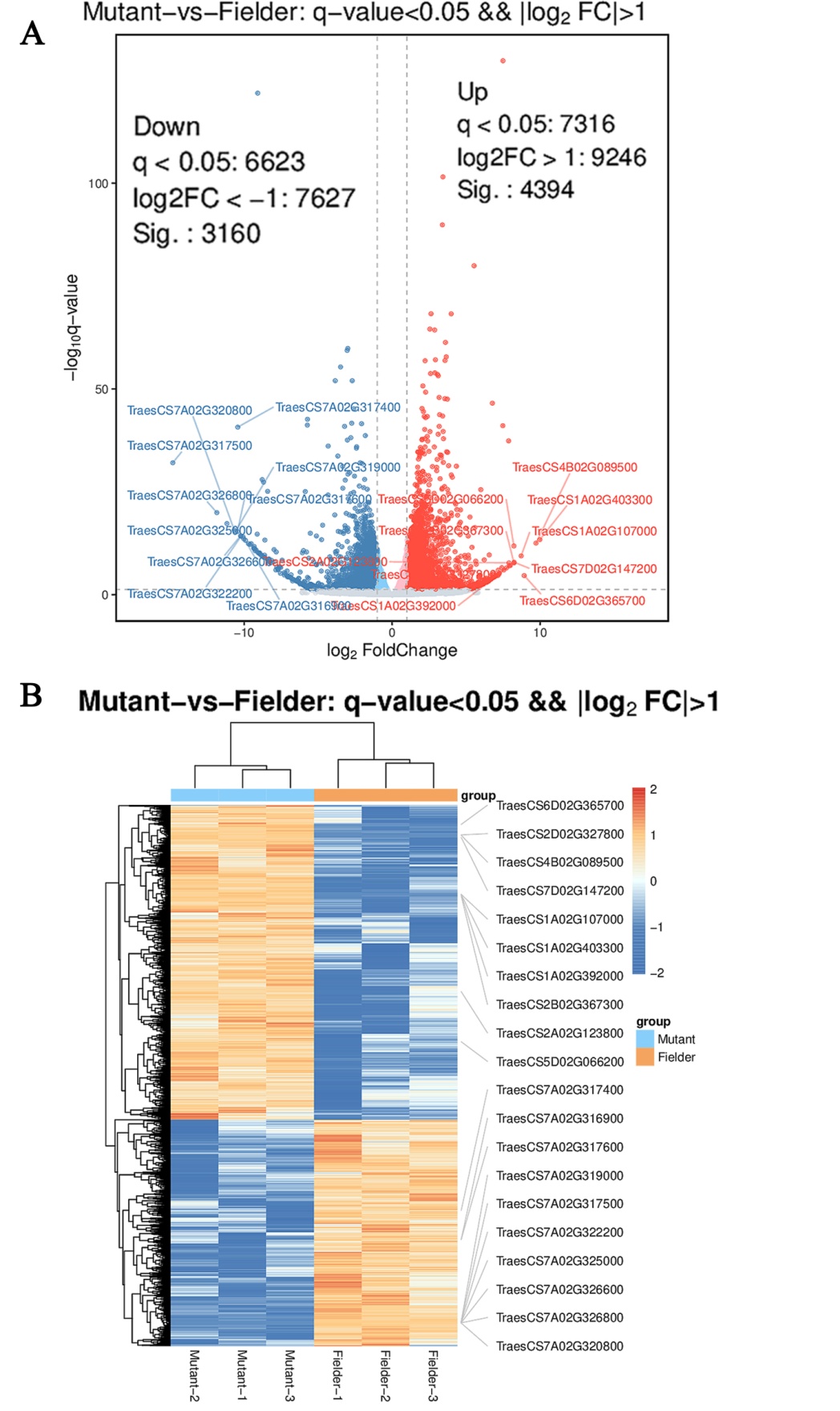


**Supplementary Figure S4 Analysis of the DEGs of the RNA-seq.**

A Volcano map of the DEGs. The red and blue dots represent upregulated and downregulated DEGs, respectively. The horizontal axis represents the log-ratio (gene expression fold change) and the vertical axis represents the probability for each gene of being differentially expressed.

B Cluster heat map of the DEGs.


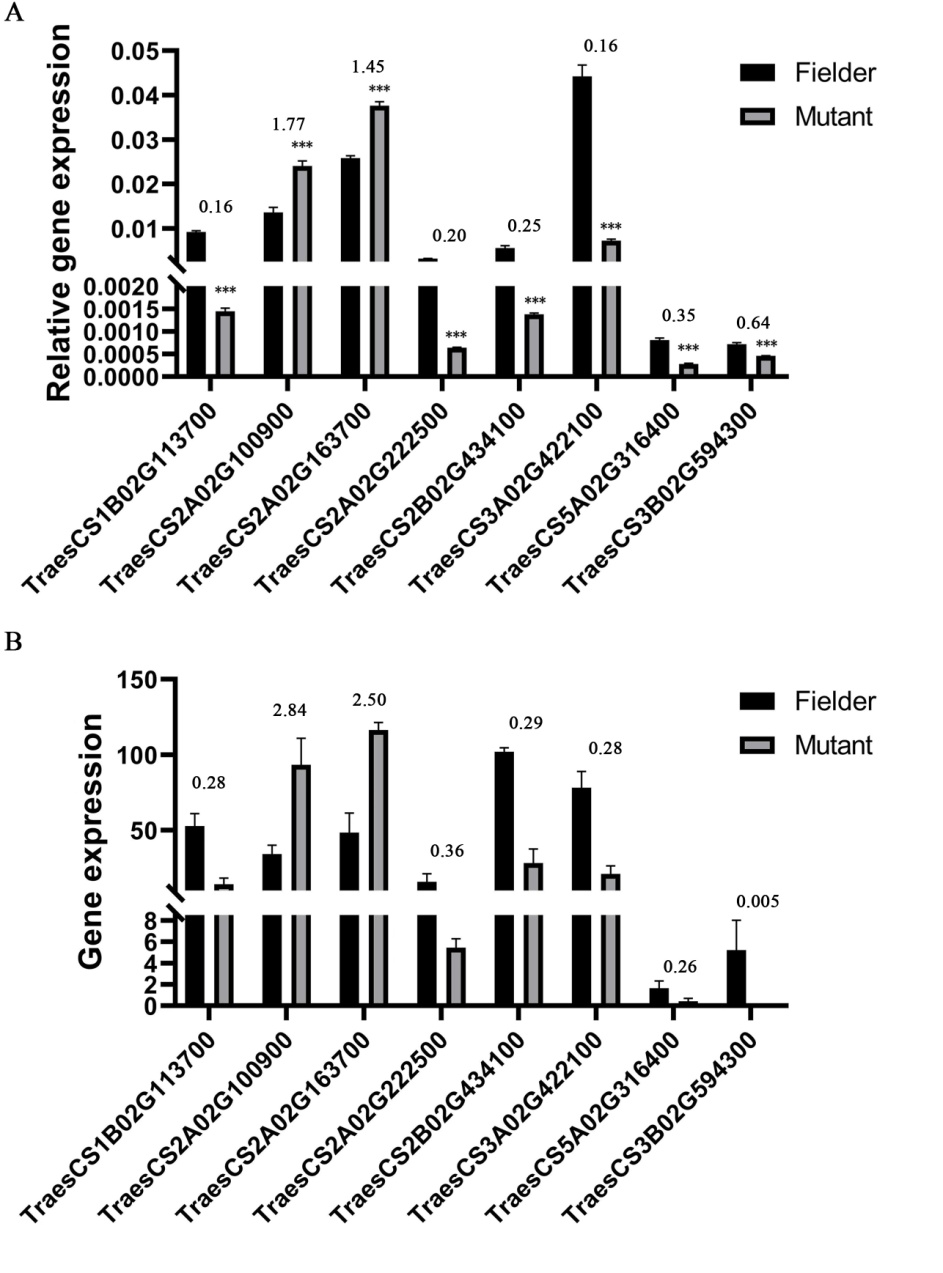


**Supplementary Figure S5 qRT-PCR analysis of the expression of the selected DEGs.**

A Relative gene expression levels analyzed by qRT-PCR. Error bars indicate standard errors of three biological replicates. Asterisks indicate a significant difference from the control value (***, *P* < 0.01; Student's *t*-test). The error bars indicate the standard deviation of triplicate values. The numbers above the columns are the values of fold-change of gene expression.

B Relative gene expression levels analyzed by RNA-seq. The error bars indicate the standard deviation of triplicate values. The numbers above the columns are the values of fold-change of gene expression.
