# Supplementary material for: Genome-edited TaTFL1-5 mutation decreases tiller and spikelet numbers in common wheat
Source: Front Plant Sci. 2023 Feb 21;14:1142779. doi: 10.3389/fpls.2023.1142779 (PMC9989183; doi:10.3389/fpls.2023.1142779)
Supplement: Supplementary file 2 [file Table_1.docx]

**Table S1 Primers used in this study.**

| Primer name | Primer sequence (5' to 3') | Melting temperature | Application |
| --- | --- | --- | --- |
| *TaTFL1*-5s-F | AATAATGGTCTCAGGCGGGAACCTCTTATTGTGGGGC | 58℃ | Knockout vectors construction |
| *TaTFL1*-5s-F0 | GGGAACCTCTTATTGTGGGGCGTTTTAGAGCTAGAAATAGC | 58℃ |  |
| *TaTFL1*-5s-R | CTGATGGGTAGAGCTCATGGCGCTTCTTGGTGCC | 58℃ |  |
| *TaTFL1*-5s-R0 | ATTATTGGTCTCTAAACCTGATGGGTAGAGCTCATGG | 58℃ |  |
| OsU3-FD3 | GACAGGCGTCTTCTACTGGTGCTAC | 58℃ | Colony PCR and sequencing |
| *TaU3*-RD | CTCACAAATTATCAGCACGCTAGTC | 58℃ |  |
| *TaU3*-FD | TTAGTCCCACCTCGCCAGTTTACAG | 58℃ |  |
| *TaU3*-FD2 | TTGACTAGCGTGCTGATAATTTGTG | 58℃ |  |
| *bar*-F | AAGCACGGTCAACTTCCGTA | 58℃ | Detecting the *bar* gene |
| *bar*-R | GAAGTCCAGCTGCCAGAAAC | 58℃ |  |
| *Cas9*-F | ACGACGATGACCTGGATAACCTCC | 58℃ | Detecting the Cas9 gene |
| *Cas9*-R | ATGCCCTCGGTCACATACTTCAC | 58℃ |  |
| JD-*TFL1*-5AF | ACTTGCAAAACCAAATATTC | 53℃ | Amplifying the target region of *TaTFL1-5A* |
| JD-*TFL1*-5AR | CGTAACATATGACAAAATGGCTCCCAT | 53℃ |  |
| JD-*TFL1*-5BF | GTGTGCTCTCCAAAAAAAAA | 62℃ | Amplifying the target region of *TaTFL1-5B* |
| JD-*TFL1*-5BR | CGTGACATATATGACAAAATGGCTCC | 62℃ |  |
| JD-*TFL1*-5DF | GCAAAAGCTAAAGTGGCAAA | 58℃ | Amplifying the target region of *TaTFL1-5D* |
| JD-*TFL1*-5DR | TGTAACATATATGACAAAATGGCTCCA | 58℃ |  |
| DL-*TFL1*-5AF | TGTCGCATGCTGGTTCCAT | 58℃ | Quantification of endogenous gene *TaTFL1-5A* |
| DL-*TFL1*-5AR | TTGTTCTATGAGGTGAC | 58℃ |  |
| DL-*TFL1*-5BF | GCTATGAGAGCCCAAAGCCG | 58℃ | Quantification of endogenous gene *TaTFL1-5B* |
| DL-*TFL1*-5BR | GCGGGTGTTGAAATGATCC | 58℃ |  |
| DL-*TFL1*-5DF | TTGTGGTTCCAAGTCGTGT | 58℃ | Quantification of endogenous gene *TaTFL1-5D* |
| DL-*TFL1*-5DR | CGAAAAGGTGCACATAACT | 58℃ |  |
| YXB-*TFL1*-5AF | CACCAGACCAAAATACCTCCCT | 58℃ | Amplification of subcellular localization sequences |
| YXB-*TFL1*-5AR | AGCGCCTCCTGGCAGCAGT | 58℃ |  |
| YXB-*TFL1*-5BF | CACCACCTCCCTCCCCTTCCAACC | 58℃ | Amplification of subcellular localization sequences |
| YXB-*TFL1*-5BR | AGCGCCTCCTGGCAGCAGT | 58℃ |  |
| YXB-*TFL1*-5DF | CACCGACGCTCATACTCTAGCAATGC | 58℃ | Amplification of subcellular localization sequences |
| YXB-*TFL1*-5DR | AGCGCCTCCTGGCAGCAGT | 58℃ |  |
| YW-*TFL1*-5F | ATGTCTAGGTCTGTGGAA | 58℃ | Amplification of in situ hybridization sequences |
| YW-*TFL1*-5R | TCAGCGCCTCCTGGCAGC | 58℃ |  |
| Sense-*TFL1*-5F | GAATTGTAATACGACTCACTATAGGGATGTCTAGGTCTGTGGAA | 58℃ | Sense probe in situ hybridization |
| Sense-*TFL1*-5R | TCAGCGCCTCCTGGCAGC | 58℃ |  |
| Anti-*TFL1*-5F | ATGTCTAGGTCTGTGGAA | 58℃ | Antisense probe in situ hybridization |
| Anti-*TFL1*-5R | GAATTGTAATACGACTCACTATAGGGTCAGCGCCTCCTGGCAGC | 58℃ |  |
| JDTB1-*TFL1*-5F | CCCCAACTCTGCCAATTA | 58℃ | Amplification of potential off-target site 1 |
| JDTB1-*TFL1*-5R | CATACACAGTATGTACCGCACG | 58℃ |  |
| JDTB2-*TFL1*-5F | CTACCTTGTTTGCCGCAA | 58℃ | Amplification of potential off-target site 2 |
| JDTB2-*TFL1*-5R | CTGGGGCTCGTTCAATCT | 58℃ |  |
| DLYZ-AF | GTACGCGTCGCTGGTGGAT | 58℃ | Quantification of endogenous gene *TraesCS1B02G113700* |
| DLYZ-AR | CGTTCTTGTCTTCACTCCTCTGG | 58℃ |  |
| DLYZ-BF | CATGGATGCCATTGCCC | 58℃ | Quantification of endogenous gene *TraesCS2A02G100900* |
| DLYZ-BR | CTGTACAGAAATGGCCGGG | 58℃ |  |
| DLYZ-CF | CCGACAAGAAGAAGAGGATTGCA | 58℃ | Quantification of endogenous gene *TraesCS2A02G163700* |
| DLYZ-CR | CTTGGTAGCAGCAGAAAGCTTG | 58℃ |  |
| DLYZ-DF | CTGCTCGGAATCAGAAAAAAAA | 58℃ | Quantification of endogenous gene *TraesCS2A02G222500* |
| DLYZ-DR | TCTCGCTTCTCATGAACTCGC | 58℃ |  |
| DLYZ-EF | GAGCCCCGCAAGAAGTCC | 58℃ | Quantification of endogenous gene *TraesCS2B02G434100* |
| DLYZ-ER | GCCCTTGATGGCGAGAGTC | 58℃ |  |
| DLYZ-FF | CGACTTCTTTGTGGATTTTGGC | 58℃ | Quantification of endogenous gene *TraesCS3A02G422100* |
| DLYZ-FR | GGATCTCCAGCTGCATA | 58℃ |  |
| DLYZ-GF | CGACTTCTTTGTGGATTTTGGC | 58℃ | Quantification of endogenous gene *TraesCS5A02G316400* |
| DLYZ-GR | GGATCTCCAGCTGCATA | 58℃ |  |
| DLYZ-HF | CCTCGTCCCCCTCCTCG | 58℃ | Quantification of endogenous gene *TraesCS3B02G594300* |
| DLYZ-HR | GCCGACTTGTCCCCGC | 58℃ |  |
| *TaActin*-F | AGTCGAGAACGATACCAGTAGTACGA | 58℃ | Quantification of reference gene |
| *TaActin*-R | GCCATGTACGTCGCAATTCA | 58℃ |  |
| *TaTubulin*-F | GATGCAGCCAACAACTTCGCC | 58℃ | Quantification of reference gene |
| *TaTubulin*-R | CAGTTCCACCTCCAACAGCGT | 58℃ |  |
