# Supplementary material for: Genome-edited TaTFL1-5 mutation decreases tiller and spikelet numbers in common wheat
Source: Front Plant Sci. 2023 Feb 21;14:1142779. doi: 10.3389/fpls.2023.1142779 (PMC9989183; doi:10.3389/fpls.2023.1142779)
Supplement: Supplementary file 3 [file Table_2.docx]

Table S2 Gene names used for the phylogenetic tree construction.

| **Species** | **Gene name** | **Gene ID** |
| --- | --- | --- |
| [*Arabidopsis thaliana*](http://plants.ensembl.org/Arabidopsis_thaliana/Info/Index) | *AtTFL1* | [AT5G03840](https://www.arabidopsis.org/servlets/TairObject?id=131459&type=locus) |
| *Hordeum vulgare* | *HvTFL1* | HORVU5Hr1G042230.3 |
| *Hordeum vulgare* | *HvTFL1a* | HORVU2Hr1G072750.1 |
| *Hordeum vulgare* | *HvTFL1b* | HORVU4Hr1G078770.1 |
| *Triticum aestivum* | *TaTFL1-U* | TraesCSU02G202000.1 |
| *Triticum aestivum* | *TaTFL1-2B* | TraesCS2B02G310700.1 |
| *Triticum aestivum* | *TaTFL1-2D* | TraesCS2D02G292000.1 |
| *Triticum aestivum* | *TaTFL1-4A* | TraesCS4A02G409200.1 |
| *Triticum aestivum* | *TaTFL1-4B* | TraesCS4B02G307600.1 |
| *Triticum aestivum* | *TaTFL1-4D* | TraesCS4D02G305800.1 |
| *Triticum aestivum* | *TaTFL1-5A* | TraesCS5A02G128600.1 |
| *Triticum aestivum* | *TaTFL1-5B* | TraesCS5B02G127600.1 |
| *Triticum aestivum* | *TaTFL1-5D* | TraesCS5D02G136300.1 |
| *Oryza sativa* Japonica Group | *RCN1* | Os11t0152500-01 |
| *Oryza sativa* Japonica Group | *RCN2* | Os02t0531600-01 |
| *Oryza sativa* Japonica Group | *RCN3* | Os12t0152000-00 |
| *Oryza sativa* Japonica Group | *RCN4* | Os04t0411400-01 |
| [*Zea mays*](http://plants.ensembl.org/Zea_mays/Info/Index) | *ZmZCN1* | Zm00001d044705_T001 |
| [*Zea mays*](http://plants.ensembl.org/Zea_mays/Info/Index) | *ZmZCN2* | Zm00001d050649_T001 |
| [*Zea mays*](http://plants.ensembl.org/Zea_mays/Info/Index) | *ZmZCN3* | Zm00001d023420_T001 |
| [*Zea mays*](http://plants.ensembl.org/Zea_mays/Info/Index) | *ZmZCN4* | Zm00001d003804_T001 |
| [*Zea mays*](http://plants.ensembl.org/Zea_mays/Info/Index) | *ZmZCN5* | Zm00001d025346_T001 |
| [*Zea mays*](http://plants.ensembl.org/Zea_mays/Info/Index) | *ZmZCN6* | Zm00001d052537_T001 |
| *Solanum lycopersicum* | *SlSP9D* | Solyc09g009560.2.1 |
| *Solanum lycopersicum* | *SlSP* | Solyc06g074350.3.1 |
| *Nicotiana attenuata* | *NaTFL1a* | A4A49_33592 |
| *Nicotiana attenuata* | *NaTFL1b* | A4A49_11462 |
| *Gossypium raimondii* | *GrTFL1a* | B456_006G155800 |
| *Gossypium raimondii* | *GrTFL1b* | B456_009G403800 |
| *Medicago truncatula* | *MtTFL1a* | MTR_1g060190 |
| *Medicago truncatula* | *MtTFL1b* | MTR_7g104460 |
