# Supplementary material for: Genome-edited TaTFL1-5 mutation decreases tiller and spikelet numbers in common wheat
Source: Front Plant Sci. 2023 Feb 21;14:1142779. doi: 10.3389/fpls.2023.1142779 (PMC9989183; doi:10.3389/fpls.2023.1142779)
Supplement: Supplementary file 4 [file Table_3.docx]

**Table S3 Sequencing data before and after quality assessment and data filtering.**

| Sample | Raw Reads | Clean Reads | Matched with reference genomes |
| --- | --- | --- | --- |
| Fielder-1 | 40570382 | 38898378 | 95.88% |
| Fielder-2 | 44066920 | 42387096 | 96.19% |
| Fielder-3 | 48768926 | 46797592 | 95.96% |
| Mutant-1 | 46400808 | 44546165 | 96.00% |
| Mutant-2 | 42764121 | 42764121 | 95.76% |
| Mutant-3 | 44183007 | 44183007 | 95.91% |
